# Strong reproductive isolation despite occasional hybridization between a widely distributed and a narrow endemic *Rhododendron* species

**DOI:** 10.1038/srep19146

**Published:** 2016-01-11

**Authors:** Yong-Peng Ma, Wei-Jia Xie, Wei-Bang Sun, Tobias Marczewski

**Affiliations:** 1 Key Laboratory for Plant Diversity and Biogeography of East Asia, Kunming Institute of Botany, Chinese Academy of Sciences, Kunming 650201, Yunnan, PR China; 2 Flower research institute, Yunnan Academy of Agriculture Sciences, Kunming 650201, PR China

## Abstract

Reproductive isolation (RI) plays an important role for speciation, but assessing reproductive barriers at all life-cycle stages remains challenging. In plants, most studies addressing the topic have been focusing on herbs with short generation times. The present study attempted to quantify several reproductive barriers between a hybridizing species pair of long-lived woody rhododendrons. Consistent with findings of previous studies, pre-zygotic reproductive barriers contributed more to total RI than post-zygotic reproductive barriers. Especially in the more widespread species geographic isolation was an important barrier, and pollinator constancy contributed exceptionally to RI in both species. Additionally to strong pre-zygotic reproductive barriers, post-zygotic reproductive barriers were considerable, and had asymmetric tendencies favoring one of the species as maternal parent. Overall, despite occasional hybridization, the present study provides evidence for strong RI between *R. cyanocarpum* and *R. delavayi*.

A still unresolved question in speciation is to trace the mode and speed of the evolution of barriers to gene flow^1^,^2^. Therefore, comprehensive analyses of mechanisms preventing gene flow between populations of sexually reproducing organisms are crucial for understanding how biological species arise and how they are maintained.

Speciation can be considered as a continuous process^3^, and if a species pair has proceeded from the initial stages of divergence, multiple reproductive barriers would be expected to have emerged^4^. Thus, studying a limited number of barriers for a species pair may skew our view of the relative importance of these barriers for certain reproductive stages^5^. Different isolating barriers act in a hierarchical order^6^, and therefore, early barriers can contribute more to total isolation than late barriers. For example a geographical barrier has to be overcome before any barriers related to pollination start playing a role. With regards to their timing in life history, barriers are usually classified using the zygote as a reference point. Hence barriers contributing to reproductive isolation (RI) can be classified as pre- and post-zygotic^5^. In recent years more attempts have been made to identify all barriers that contribute to RI, however, the prevalence of herbs in these studies is still considerable^6^,^7^. This might be due to this type of assessment being much more challenging for woody plants as opposed to annual plants because of issues arising from the long generation times involved, and hence problems identifying barriers related to later stages in life-history (e.g. between seedling establishment and reaching reproductive age). This is one of the main reasons why we still have very little knowledge about RI in trees and tropical taxa, when compared to temperate herbs^7^.

The genus *Rhododendron* is the largest genus in the family Ericaceae, containing approx. 1000 species, all of which are woody shrubs or trees. Subgenus *Hymenanthes*, one of its eight recognized subgenera, comprises 225 species in 24 subsections^8^,^9^, with all of the investigated subgeneric species being diploids (2n = 26)^9^. Generally reproductive barriers in the genus are weak, especially between species belonging to the same subsection, which is evidenced by several reports of natural hybrids in the wild^10^,^11^,^12^,^13^,^14^. Furthermore, even hybrids between less closely related species are often obtainable with ease, which resulted in a large amount of artificial hybrids for ornamental purposes, undoubtedly contributing to the popularity of the genus in horticultural circles. It has been hypothesized that most members of the genus in the Sino-Himalayan region originated from a relatively recent radiation event triggered by the uplift of the Himalayas^15^. This could be one possible factor for the overall high reproductive compatibility between members of the genus. However, how much other factors, as for example long generation time or demography contributed is completely unknown. Furthermore, most studies in the genus have focused on a small subset of reproductive barriers, such as hybrid swarm composition^11^,^13^,^14^,^16^, and genetic incompatibilities^14^. Hence, to date no comprehensive study considering all possible stages potentially conveying reproductive barriers between any two members of the genus has been published.

A previous study of hybrids between the two species *Rhododendron delavayi and R. cyanocarpum*, found an exceptionally high number of F_1_ and F_2_ hybrids in a contact zone, and provided evidence for asymmetric backcrossing towards *R. delavayi*^11^. Relatively recent habitat disturbance of the area in the 1950 s was put forward as one possibility for the emergence of this hybrid zone^12^. However, as ecological data for the species was lacking, it remained unclear how factors such as seed viability and seedling establishment contributed to the unusual pattern, and how strong reproductive isolation between the species remained despite the occurrence of hybrids.

Therefore, the specific goal of the present study was to conduct a comprehensive assessment of diverse barriers that can contribute to RI (see table 1). The questions we wanted to answer were (1) How strong are the different reproductive barriers, and what is their relative contribution to overall RI? (2) Do some of these barriers act asymmetrically, providing an explanation for the observed asymmetric hybridization patterns? (3) Is there evidence for ecological selection during seed germination with regards to soil type, hence are hybrid seeds affected differently depending on location?

## Results

### Geographic isolation

In total, 553 specimens of *R. delavayi* were listed on the website of the Chinese virtual herbarium, and 83 specimens of *R. cyanocarpum*. The *R. delavayi* specimens represented records from 56 counties, of which 43 were counties of Yunnan province, eight counties of Guizhou province, three counties of Tibet, and two counties of Guangxi province. All the specimens of *R. cyanocarpum* were from Cangshan mountain, confirming its restricted distribution. Therefore, for R. delavayi RIgeograph = 1 − (1/56) = 0.98 whereas RIgeograph = 0 for R. cyanocarpum as the only location where it was recorded was sympatric with R. delavayi.

### Phenological Isolation

In 2008, *R. cyanocarpum* flowered from 18^th^ of February to 19^th^ of May, whereas the flowering period of *R. delavayi* lasted from 25^th^ of February to 2^nd^ of June. Therefore, *R. cyanocarpum* flowered for 92 days (February in 2008 had 29 days) and *R. delavayi* flowered for 99 days; for 85 days *R. cyanocarpum* and *R. delavayi* were flowering at the same time. Hence RI_phenology_ = 1 − (85/92) = 0.043 for *R. cyanocarpum*, and RI_phenology_ = 1 − (85/99) = 0.141 for *R. delavayi*.

### Floral Isolation

#### Nectar composition

**Figure 1:**
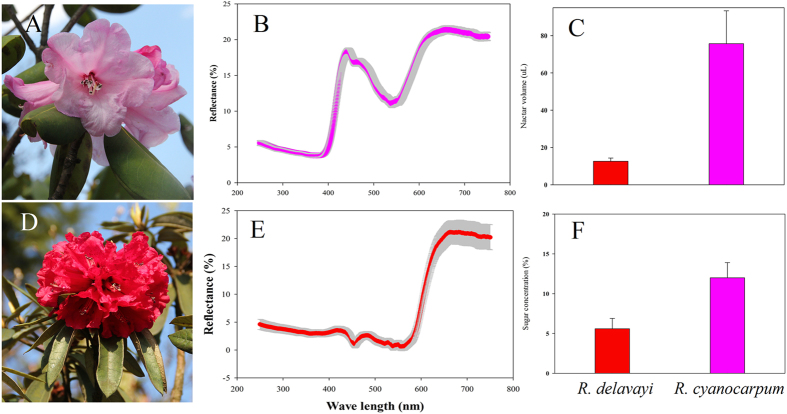
Flowers with its petal color reflectance, nectar volume and sugar concentration of *R. cyanocarpum* and *R. delavayi*. (**A**) *R. cyanocarpum* flower. (**B**) Petal color reflectance of *R. cyanocarpum*. (**C**) Nectar volume. (**D**) *R. delavayi* inflorescence. (**E**) Petal color reflectance of *R. delavayi*. (**F**) Nectar sugar concentration.

*R. cyanocarpum* flowers contained on average 75.71 ± 17.64 ul of nectar (n = 20), while *R. delavayi* flowers contained 12.6 ± 1.75 ul (n = 20), which was significantly different (t = 19.15, p < 0.001; Fig. 1C). This large difference was mostly due to *R. cyanocarpum* having five nectar pouches, all of which produce nectar, while *R. delavayi* has one dominant nectar pouch, which is the only one producing nectar. Looking at nectar production per nectar pouch, *R. cyanocarpum* nectar pouches produced 15.14 ± 0.75 ul of nectar, which is still significantly different (t = 2.918, p = 0.006). The sugar concentration in *R. cyanocarpum* nectar was 12 ± 2% and 6 ± 1% for *R. delavayi* nectar (both n = 20), which was also significant (t = 10.51; p < 0.001; Fig. 1F).

#### Petal color reflectance

While *R. cyanocarpum* flowers showed a marked peak in the reflectance spectrum at 430 nm, this peak was completely absent in *R. delavayi* flowers (Fig. 1B,E). In addition, a peak in the reflectance spectrum above 600 nm was detected in both species.

#### Volatile compounds in flowers

In the floral extracts of *R. cyanocarpum* 21 volatile compounds were identified, accounting for 96.73% of total extracted mass, and in extracts of *R. delavayi* 18 volatile compounds were identified, accounting for 70.38% of total extracted mass (Supplementary Table 1).

Most of the detected compounds belonged to either aliphatics (accounting for 64.37% in *R. cyanocarpum*, and 56.2% in *R. delavayi*) or terpenoids (accounting for 28.88% in *R. cyanocarpum*, and 11.4% in *R. delavayi*) (Table 2). Additional quantification of produced volatile compounds showed that production of all compound groups in *R. cyanocarpum* exceeded production in *R. delavayi* by about one order of magnitude (Aliphatics: 595.98 ng/h/flower vs. 38.26 ng/h/flower; Terpenoids: 276.65 ng/h/flower vs. 7.52 ng/h/flower; Benzenoids: 22.96 ng/h/flower vs. 2.14 ng/h/flower; Table 2). If we taking the one third of flower numbers per inflorescence of RC to RD (on average 6 flowers of RC vs. 19 flowers of RD) into account, the much larger amount of compounds were still examined in *R. cyanocarpum* than in *R. delavayi* per inflorescence (Aliphatics: 3575.88 ng/h/inflorescence vs. 726.94 ng/h/inflorescence; Terpenoids: 1659.9 ng/h/inflorescence vs. 142.88 ng/h/inflorescence; Benzenoids: 137.76 ng/h/inflorescence vs. 40.66 ng/h/inflorescence; Table 2).

### Pollinator mediated isolation

In 2008, 32 bumblebees and 1 butterfly were recorded visiting flowers of *R. cyanocarpum*; 39 bumblebees and 8 wasps were recorded visiting *R. delavayi* flowers. As experimental plots for pollinator constancy had not been set up in 2008, RI was calculated for pollinator assemblage only. RI due to shared pollinators (bumblebees) for *R. cyanocarpum* = 1 − (31/32) = 0.031; and RI = 1 − (39/47) = 0.17 for *R. delavayi*.

In 2011, observations were carried out in three plots: plot-1 (*R. cyanocarpum* dominated population); plot-2 (*R. delavayi* dominated population); plot-3 (hybrid zone). Over the course of the observation time, 30 bumblebees were recorded visiting flowers of *R. cyanocarpum* (plot-1: 28, plot-2: 2, plot-3: 0), and 11 bumblebees visiting flowers of *R. delavayi* (plot-1: 6, plot-2: 5, plot-3: 0). Furthermore, 14 visits of birds (*Yuhina occipitalis*) to flowers of *R. delavayi* were recorded (plot-1: 0, plot-2: 14, plot-3: 0). During the whole of the observation time there were no pollinator visits to plot-3. Although bumblebees visited both species, we never witnessed a transfer visitation between flowers of the two species in any of the plots. Therefore, reproductive isolation for pollinator assemblage was relatively weak (RI for *R. cyanocarpum* = 1 − (30/30) = 0; and RI = 1 − (11/25) = 0.56 for *R. delavay*i) in comparison to RI resulting from pollinator constancy: RI = 1 for both species.

### Pollen tube growth

For all four pollination treatments the pollen tubes had grown a very short distance 24 hours after application of the pollen to the stigmas. With *R. cyanocarpum* as maternal species, pollen tubes from intra-specific crosses had on average grown 12.32 ± 1.3% of the total length of the style, whereas pollen tubes from inter-specific pollination reached only 2.2 ± 0.3% of total style-length. This difference is highly significant (t = 7.66, p < 0.001). When *R. delavayi* was the maternal species, pollen tubes from intra-specific pollinations had reached 6.1 ± 2.0% of the total style-length, compared to 2.7 ± 2.0% of total style-length for inter-specific pollinations. This difference was also highly significant (t = 11.41, p < 0.001). As no pollen tubes had reached the ovules, only a tentative RI can be estimated. Making the very simplified assumption that the difference in pollen tube growth at the measured stage reflects the likelihood for later fertilization, RI due to conspecific pollen precedence for *R. cyanocarpum* = 1 − (2.2/12.32) = 0.821, and RI = 1 − (2.7/6.1) = 0.557 for *R. delavayi*.

### Seed production

**Figure 2:**
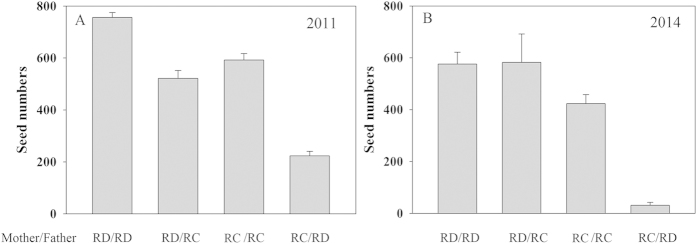
Seed production from inter- and intra-specific pollinations in 2011 (A) and 2014 (B). RD: *R. delavayi*; RC: *R. cyanocarpum*. The error bars indicate S. E.

All pollination treatments resulted in seed-set (Fig. 2A,B). Although the number of seeds produced was significantly affected by years (F = 5.03; p = 0.027), there were no significant interactions between year and the other factors (Table 3). Furthermore, pollen source (F = 21.33, p < 0.001) and maternal species (F = 29.58, p < 0.001) had a significant effect on seed number, with a significant interaction between these two factors (F = 6.2, p = 0.014). RI_seed production_ for *R. cyanocarpum* = 1 − (223.2/592.6) = 0.623 and RI_seed production_ for *R. delavayi* = 1 − (521.8/756) = 0.310 in 2011, respectively. In 2014, RI_seed production_ for *R. cyanocarpum = *1 − (31.06/423.59)* = *0.927 and RI_seed production_ for *R. delavayi* = 1 − (582.33/576.12) = −0.011 (Fig. 2A,B).

### Soil physico-chemical analysis

With the exception of available potassium, all physico-chemical soil properties showed significant differences between the three sites (Table 4). In measures of pH, soil organic matter, and total potassium the hybrid site is intermediate between the two parental species dominated sites (Table 4). However, measures of total nitrogen and total phosphorus are significantly higher for the hybrid site than for either parental site. Available phosphorous was comparable between the hybrid site and the *R. cyanocarpum* dominated site, but both were significantly higher than the *R. delavayi* dominated site (Table 4).

### Seed germination

#### Medium: distilled water

**Figure 3:**
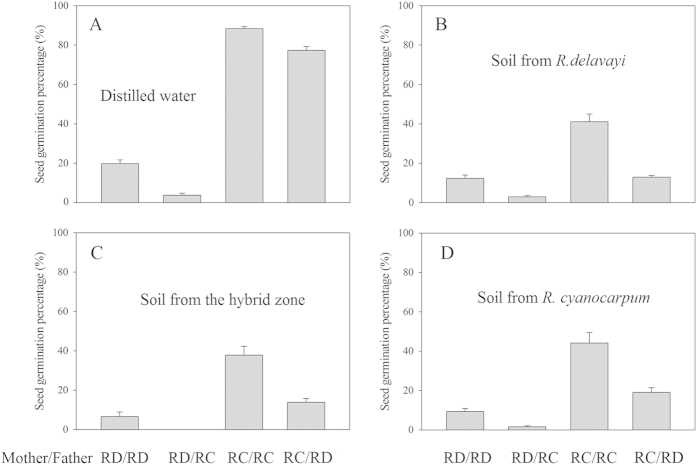
Seed germination percentage under distilled water and three soil treatments. (**A**) Distilled water. (**B**) Soil samples collected from *R. delavayi* population. (**C**) Soil samples collected from the hybrid zone. (**D**) Soil samples collected from *R. cyanocarpum* population. RD: *R. delavayi*; RC: *R. cyanocarpum*. The error bars indicate S. E.

When seeds were germinated on filter paper soaked with distilled water, seed germination rate was higher for seeds obtained from *R. cyanocarpum* plants when compared to seeds from *R. delavayi* plants (F = 1060, p < 0.001; Table 5, Fig. 3A). In addition, pollen source also had a significant effect on seed germination (F = 38.15, p < 0.001), with the interaction term between the two factors being not significant (Table 5). For seed obtained from *R. cyanocarpum*, the germination rate was 0.883 ± 0.01 for seeds from intra-specific pollinations compared to 0.773 ± 0.019 for seeds from inter-specific pollinations (Fig. 3A). For seed from *R. delavayi*, the germination rate was 0.197 ± 0.019 for seeds from intra-specific pollination compared to 0.037 ± 0.01 for seeds from inter-specific pollination (Fig. 3A). Thus RI_seed germination_ of *R. cyanocarpum* = 1 − (0.773/0.883) = 0.125 while RI_seed germination_ of *R. delavayi* = 1 − (0.037/0.197) = 0.812 (Fig. 3A).

#### Medium: different soil types

Compared to germination using distilled water, seed germination in soil overall was significantly reduced (F = 307.99, p < 0.001; Table 5). However, between the three different soil types no significant differences in germination rate were detected (F = 2.31, p = 0.115). On the other hand, the pollen source and mother species had both a significant effect on germination rate (pollen source: F = 83.81, p < 0.001; mother species: F = 211.7, p < 0.001), with their interaction term being significant as well (F = 39.38, p < 0.001).

Because the soil type had no significant effect on seed germination, we averaged the three different soil treatments. Taking the mean of all three soil treatments, the germination rate for seed from *R. cyanocarpum* was 0.41 ± 0.018 for seeds from intra-specific pollinations compared to 0.153 ± 0.019 for seeds from inter-specific pollinations. For seed from *R. delavayi*, the germination rate was 0.095 ± 0.017 for seeds from intra-specific pollination compared to 0.015 ± 0.009 for seeds from inter-specific pollination. Thus RI_seed germination_ of *R. cyanocarpum* = 1 − (0.153/0.41) = 0.627 while RI_seed germination_ of *R. delavayi* = 1 – (0.015/0.095) = 0.833.

## Discussion

Our assessment of multiple reproductive barriers indicated that *R. cyanocarpum* and *R. delavayi* are nearly completely reproductively isolated (overall RI = 1 for pre-pollination stages, and overall RI = 0.94 for *R. cyanocarpum* and 0.937 for *R. delavayi* in post-pollination stage (Table 6).

### Prezygotic barriers

#### Geographic isolation

Geographic isolation protects most individuals of *R. delavayi* completely from geneflow originating from *R. cyanocarpum*, while on the other hand all known plants of *R. cyanocarpum* grow at least in parapatry with *R. delavayi*, and hence may hybridize with it. This results in a diametrically opposite contribution of geographic isolation to RI in the two species, and while it is the most important barrier for *R. delavayi* (RI = 0.98) it is non-existent in *R. cyanocarpum* (RI = 0). This should apply to any case for which one species of a hybridizing species-pair is a narrow endemic, which should then have by default a lower barrier to geneflow than the wide-spread species. However, this only applies to the species as a whole, and the situation in sympatry is likely to be different in each case.

#### Isolation due to phenology and pollinators

Phenology and pollinator assemblage conveyed a higher RI for *R. delavayi* than for *R. cyanocarpum*. Both barriers were negligible in *R. cyanocarpum* (RI_phenology_ = 0.043, RI_pollinator assemblage_ = 0), while considerable in *R. delavayi* (RI_phenology_ = 0.141, RI_pollinator assemblage_ = 0.56). However, pollinator behavior contributed the strongest barrier in sympatry for both species (RI_pollinator behavior_ = 1). It is obvious that the estimate of RI = 1 for pollinator behavior, meaning a complete barrier, is flawed, as hybrids do occur in the wild, but nonetheless, pollinator constancy is probably a crucial barrier for this species pair. A just-published work of quantifying RI in sympatric woody species of *Cyrtandra* (Gesneriaceae) revealed strong RI in post-zygotic stages, but reproductive barriers mediated from pollinators that were proved to be important by our current study, were neglected^17^.

The relatively high RI for the pollinator assemblage in *R. delavayi* (0.56) was due to birds visiting the flowers, while no birds were observed visiting *R. cyanocarpum* flowers. That *R. delavayi* flowers have floral characteristics indicative of ornithophily is also supported by the floral analyses, as their nectar had a low sugar concentration^18^, overall lower emission of floral volatile compounds, and were lacking the characteristic peak at 430 nm in the reflectance spectrum, which has been shown to be attractive for insects^19^. However, despite the evidence for ornithophily, *R. delavayi* flowers were frequently visited by bumblebees (a total of 58 visitations by bees in 2008 and 2013).

Flowers of *R. cyanocarpum* on the other hand had many features which are generally observed in insect pollinated plants. They possessed a characteristic peak at 430 nm in the reflectance spectrum and showed overall high emissions of volatile compounds rich in aliphatics and terpenoids which are often attributed to insect attraction^20^. Previous pollinator observations showed that *R. cyanocarpum* flowers do indeed attract several different groups of insects, but only bumblebees were ever observed to effect pollination^21^. The reason that we only recorded bumblebees in the present study is probably due to the circumstance that observations were carried out in spring, early in the flowering season. In the study area both species grow at relatively high altitudes (>2800 m) where temperatures are quite low in spring, and pollinators that can cope with colder conditions, such as bumblebees, are therefore likely to be overrepresented in the current data. However, as they are the only reported effective pollinators, this bias should have a minor effect on pollinator assemblage.

The largest barrier in sympatry was pollinator constancy, as no transfer visits were observed, which indicates that cross-pollination is a relatively rare occurrence. It would have been interesting to ascertain this behavior also in the hybrid zone, as only pollinator visitation data for the two parent-dominated plots (plot-1, plot-2) could be obtained. Although a plot was established in the hybrid zone (plot-3), not a single pollinator was attracted to the flowers. Other studies have, however, reported that the occurrence of hybrids can facilitate pollen transfer between parental species^22^. It also remains unclear if the experimental setup could have lead to increased constancy, for example the preferred flower was always available in close proximity, which would not be the case if a bumblebee crossed the border from a site dominated by one of the species into a larger patch dominated by the other species. Hence, in certain settings accidents might occur more often than the data suggest.

#### Pollen precedence

The RI conveyed by pollen precedence was estimated to be high for both species (0.821 and 0.557), but these values are only tentative, as only data from the first 24 hours was available, and at that stage the furthest some pollen tubes had grown was about 12% of the distance to the ovules. Furthermore, it is unclear how often pollen from both species is transferred to the same stigma under natural conditions, as frequently several flowers in one inflorescence remain un-pollinated, indicating that several flowers may receive pollen scarcely, thus making it less probable for the pollen from the different species getting to the same stigma at the same time.

### Post-zygotic barriers

The amount of hybrid seed produced and its viability were affected differently in the two species. While *R. cyanocarpum* produced significantly fewer seeds when the pollen source was heterospecific (492 vs. 125), the picture was less clear for *R. delavayi*. In 2011 there was a noticeable effect on hybrid seed number (756 vs. 522), but in 2014 there was no significant difference between numbers of seed obtained from the two pollination treatments (576 vs. 582). Thus in a scenario most favorable for hybridization there is no evidence for RI resulting from a reduction in seed in *R. delavayi*.

However, RI resulting from a difference in seed germination was overall high in *R. delavayi*, with no significant difference between germination in water or soil (RI > 0.8, Table 6). On the other hand, for hybrid seed from *R. cyanocarpum* mothers the germination rates were significantly different between these two media (0.125 vs. 0.627, Table 6). Thus, while most hybrid seed from *R. delavayi* mothers was inviable, hybrid seed from *R. cyanocarpum* mothers seemed to be mostly viable, but apparently germination was somehow negatively affected in soil. In the natural environment (soil) the RI is therefore likely to be high for both species, which would favor *R. delavayi* as mother species for hybrids, as the higher number of hybrid seeds produced counteracts the lower germination success. Genetic data suggests that this is the case, as most hybrids seem to have a *R. delavayi* chloroplast type^11^.

That a large portion of *R. delavayi* hybrid seed was inviable could perhaps partly be explained if genetic incompatibilities affecting seed-set were acting at earlier stages in *R. cyanocarpum*. If potentially inviable seeds were aborted in *R. cyanocarpum* before they are formed, but seed development proceeded in *R. delavayi* to a stage when it is difficult to distinguish viable seeds from inviable ones, the germination rate would necessarily be lower for *R. delavayi* hybrid seed. The observed lower hybrid seed production for *R. cyanocarpum* would also fit this pattern. However, even if this would be the case, it would not explain the significantly different germination rates of *R. cyanocarpum* hybrid seed in soil as opposed to water. If some form of ecological selection plays a role for RI in *R. cyanocarpum* seed germination would be an interesting question for further research, as the current data does not provide clues as to which factors might have resulted in the difference.

### Summary

For the widespread species *R. delavayi* geographic isolation is the most important barrier at the species level. In sympatry the highest contribution to RI for both species comes from pollinator constancy, with other pre-zygotic barriers being very weak in *R. cyanocarpum* but considerable in *R. delavayi*. Additionally, post-zygotic barriers are strong, and seem to be slightly asymmetric, indicating higher likelihood for occurrence of hybrid seedlings originating from *R. delavayi* mothers. With pre-zygotic barriers contributing considerably more to overall RI than post-zygotic barriers, the data fits the pattern already observed by Lowry *et al*.^23^.

However, it should be pointed out that this pattern mostly results from geographic isolation and pollinator behavior, and especially the estimate for the latter is flawed in the current study. Furthermore, as the investigated species are woody perennials with long generation times, only performance of hybrid seed in early life stages could be assessed. The fitness of hybrids that are being produced despite strong RI remains, however, unclear.

## Materials and Methods

### Study species

*Rhododendron delavayi* is a widely distributed species in southwest China, generally growing at altitudes below 3000 m. Members of the genus have been reported in the provinces of Yunnan and Guizhou, and further west reaching into some areas of Tibet. *R. cyanocarpum*, on the other hand, is a narrow endemic which is only found in one location in China, Cangshan mountain, growing at altitudes above 3100 m. Due to its limited range *R. cyanocarpum* was listed in the Red List of Rhododendrons^24^. The two species can easily be distinguished by their bark, *R. delavayi* has very deeply-ridged and rough bark, whereas it is smooth in *R. cyanocarpum*; the leaves of *R. delavayi* are lanceolate and their abaxial leaf surface is covered with cream-brown hairs, which are lacking in *R. cyanocarpum* leaves that additionally are rounded. Furthermore, *R. cyanocarpum* has a big calyx in both flower and fruit, whereas it is always small in *R. delavayi*, and *R. delavayi* has deep-red flowers, while they are pink in *R. cyanocarpum*.

### Calculation of RI

For each stage-specific mechanism that we examined, the effect of that mechanism was quantified using indices of reproductive isolation (RI). With reference to Kay^5^, each RI value was generally varying from zero to one, with zero representing no barrier to inter-specific gene flow and one representing a complete barrier (Table 1). In the rare case of negative values, they would be indicative of stages at which hybridization is favored.

### Geographic isolation

To assess and quantify geographical overlap of species is generally problematic, as for a wider range, units will have to be chosen more or less arbitrarily^7^. Due to the type of records we used (herbarium specimens), we chose to use counties as units to check for co-occurrence of the two species. This is a very coarse-grained approach, but specimen information in the area was expected to be too scarce to merit an approach using a finer, custom, grid.

Specimen information for *R. delavayi* and *R. cyanocarpum* was obtained for all relevant specimens deposited in Chinese herbaria that are accessible via the Chinese virtual herbarium (http://www.cvh.org.cn, accessed: 16 June 2015). According to locality information on the specimens counties were then identified in which the species occurs.

### Phenological Isolation

In the contact zone of the two species approximately 500 mature individuals of *R. cyanocarpum*, and 50 mature individuals of *R. delavayi* could be found. As co-flowering in the contact zone was deemed most important, and flowering generally occurred at the same time in the whole area, only this population was used to assess flowering time. Local people were hired for the relevant months (February to July) in 2008 to record weekly if individuals were flowering.

### Floral Isolation

It is common that a pollinator assemblage can vary significantly over the course of a prolonged flowering period, and also differences between years can be expected^25^,^26^. Both species can have flowering times lasting more than three months, which could therefore lead to biased pollinator assemblage assessments, if observations would only be carried out for a selected period in the flowering season. To avoid this bias, in addition to pollinator observations, we aimed at predicting general pollinator preference by distinguishing key flower characteristics. To this purpose we measured nectar volume and sugar concentration, light reflectance spectra of petals, and extracted floral volatile compounds.

### Nectar volume and nectar sugar concentration

For measurements of nectar volume and corresponding sugar concentration, ten plants from each species were selected that were at least 10 m apart, and which had flower buds about to open the next day. From each of the 20 selected plants two flower buds about to open the next day were enclosed in plastic bags to minimize evaporation, and prevent disturbance by visitors. The following day, after the buds had opened, at around 10:00, the nectar volume was directly measured by extraction from the nectar pouches with a calibrated 100 μL capillary (Sigma Chemical Co., St. Louis, MI, USA). Sugar concentration was then obtained using a hand-held refractometer (Eclipse; Bellingham & Stanley Ltd., Basingstoke, UK), measured as grams of sugar per gram of nectar, and expressed as percentage.

### Flower petal reflectance

Two mature flowers each of ten different individuals per species were collected, and one petal per flower was used for measurements. Hence 40 petals were measured, 20 *R. delavayi* and 20 *R. cyanocarpum*. To assess light reflection patterns of petals at different wavelengths, we obtained spectral data from petals using an S2000 miniature fiber optic spectrometer with a PX-2 pulsed xenon lamp (Ocean Optics, Dunedin, FL, USA). All measurements were carried out in the range from 250 to 750 nm, using 0.38 nm increments. As changes in the reflectance pattern along the same petal were known to show negligible variation^26^, only one measurement per petal was taken.

### Floral volatile compounds

For the collection of volatile compounds, three young inflorescences per species, each from a different individual were selected. The inflorescences were taken to a close by hotel, and wilted or non-opened flowers were removed until 25 flowers remained per species, though such treatment might change the scent composition of an infloresence, since those would normally be present. The three inflorescences per species were then enclosed in Tedlar bags (Dupont, USA). Extraction was started from 12:00, which corresponds to the time of highest pollinator activity. Using a pump (inlet flow rate 300 mL/min), volatiles were then extracted for 6 h from the enclosures into cartridges containing the adsorbent Porapak Q (150 mg, mesh 60/80; Waters Associates, Inc., USA). Prior to use, the adsorbent cartridges had been cleaned with 2 ml diethyl ether and dried with nitrogen gas. For analysis the trapped volatiles were eluted with 400 μL dichloromethane and the concentration was subsequently increased by reducing the original volume 1:5 through evaporation at room temperature, aided by a gentle stream of nitrogen. Then 720 ng of n-nonane was added to each sample as standard for later quantification, and the samples were stored at −20 °C.

The samples were then run on an Agilent Technologies HP 6890 gas chromatograph, equipped with an HP-5MS column (30 m × 0.25 mm inner diam, 0.25 μm film thickness), and linked to an HP 5973 mass spectrometer. Helium was used as a carrier gas at a flow rate of 1 ml/min. Split inlet and FID were held at 250 °C. Column temperature was programmed to rise from 40 °C (5-min hold) to 250 °C (20-min hold) at 3 °C/min. The mass spectra were taken at 70 eV (in EI mode) with a scanning speed of 1 scan/s from m/z 30 to 350. Compounds were tentatively identified by comparing mass spectra and relative retention times with those of standard compounds and with the Wiley NIST 05 mass spectral database. Their relative proportions (%) were calculated by dividing the absolute amounts of individual compounds by the sum of all compounds and multiplying by 100. The absolute amounts of all samples were calculated by the internal standard method. Because some flowers were removed especially from inflorescences of *R. delavayi*, emission quantity of compounds was tentatively calculated as the absolute emission per flower multiplied by average flower numbers per inflorescence for both species.

### Pollinator mediated isolation

In April 2008, to examine the main pollinators, we recorded visitations of potential pollinators in one individual of *R. cyanocarpum* and one individual of *R. delavayi*. For each species a total of 24 h for 4 successive days in *R. cyanocarpum* dominant and *R. delavayi* dominant population were observed respectively.

In April 2011, during seven consecutive sunny days pollinator preference for the two species was observed. To assess whether insects discriminated between the species or transferred pollen between flowers of both species, three experimental plots were established, one in a population comprising predominantly *R. cyanocarpum* (plot-1), one in a *R. delavayi* dominated population (plot-2) and another one in the hybrid zone (plot-3).

In each of the plots eight flowers of each species (8 *R. cyanocarpum*, 8 *R. delavayi*) were arranged in a 4 × 4 grid structure, with flowers 0.5 m apart and alternating flowers of different species by row and column, so that row one and three started with a different species to row two and four. For the grid freshly opened flowers were collected and placed into a bottle with water. Visiting pollinators to the plots were then recorded on four sunny days in plot-1 for a total of 24 hours, and on three sunny days in plot-2 and plot-3 for a total of 18 hours of each. Only insects that were observed to visit flowers were recorded.

### Hand pollination treatments

To investigate cross-fertility of the two species, four types of hand pollination treatment were carried out employing a full orthogonal design. The same pollination treatments were carried out in two years, 2011 and 2014. Per treatment anthers of more than 30 flowers of five maternal individuals were removed before anthesis; these emasculated flowers were then bagged with netbags. After opening of the corolla, the flowers were pollinated using anthers from freshly collected flowers, and consecutively re-bagged. For cross-pollination with con-specific pollen, flowers of the same species were collected from individuals that were growing at least 10 m from the experimental plants. Fruits were harvested at the end of October, and the number of seeds was counted for each fruit.

### Pollen tube growth

To assess if differential pollen tube growth, depending on the maternal parent contributes to RI between the species, a subset of the flowers from the hand pollination treatments carried out in April, 2014 was used. For each of the four treatments, 10 flowers each from 5 flowering plants for each species were harvested 24 hours after pollen had been applied to the stigma. The length of each harvested pistil was recorded, after which it was dissected and softened by incubation in 0.1 M NaOH at 60 C for 1 h. Afterwards, the pistils were incubated in 0.1% aniline blue (pH 8.3) for 48 h. Then slides were examined under a fluorescence microscope with blue excitation (410 nm) and for each pistil the growth of pollen tubes. Prior to the observation, the length of style per treated flower was measured. Then the relative pollen tube growth rate was calculated as the ration of pollen tube length to the total length of the style.

### Soil sampling and physico-chemical analysis

In 2013, soil was obtained from three sites, one site dominated by *R. cyanocarpum*, one site where mostly *R. delavayi* plants were found and the hybrid zone. Four plots measuring about 4 × 4 m were randomly selected in each of the three sites, and prior to soil sampling, litter and organic material was removed from the soil surface. Surface soil was then collected close to several (>4) *Rhododendron* individuals growing in the plot limits and subsequently mixed, giving a total of 12 soil samples (four samples each from each of the three sites). The samples were taken to the laboratory, and after removing root material and stones, were sieved using a 2 mm mesh sieve.

Aliquots of 15–20 g were oven dried at 105 °C for 24 h, and afterwards homogenized to a saturated colloid with 0.01 M NaNO_3_. From this colloid measurements were obtained as follows: pH was measured using a pH-meter (PHS-3C; Shanghai Precision & Scientific Instrument, Shanghai, China); total phosphorus was determined colorimetrically after nitric/perchloric acid digestion; total nitrogen was determined by UV spectrophotometric measurement after nitric/perchloric alkaline digestion; total potassium was determined by atomic absorption (spectrophotometric); available phosphorus was determined by the Olsen method; soil organic matter was determined by oil bath K_2_CrO_7_ titration; available potassium was extracted with 1 N ammonium acetate and analyzed by atomic absorption (spectrophotometric); all methods were carried out as described by Hou *et al*.^27^.

### Seed germination

#### Seed germination using distilled water

Seeds obtained from each of the four cross-pollination treatments in 2014 were placed on filter paper maintained moist with distilled water and placed in Petri dishes at room temperature (~25 °C). From each of the four pollination treatments 200 seeds were used, separated into four replicates of 50 seeds, hence 800 seeds in total were used. The petri dishes were left for 30 days while insuring that they stayed moist, and after this period the number of germinated seeds was counted in each petri dish. The fraction of germinated seeds for each replicate was then calculated by dividing the number of germinated seeds by 50.

#### Seed germination in different soil types

The seeds obtained from the same treatments as mentioned above were used to also assess germination in the different soil types collected for physico-chemical analysis. For each of the three soil types, four replicates for seed obtained from each of the four pollination treatments were carried out (3 types of soil × 4 pollination treatments × 4 replicates each = 48). Petri dishes were filled to a depth of 2 cm with the respective soil, and 50 seeds were spread on the surface. The soil was then moistened with distilled water, and the petri dishes were incubated for 30 days at room temperature (~25 °C). Therefore, in total of 2400 seeds were used to do the experiment. After 30 days the number of germinated seeds was counted in each of the petri dishes, and germination rate was calculated by dividing by 50.

### Data analysis

Significance of the difference of the means for nectar volume and sugar concentration between the two species was assessed using independent two-sample t-tests. Physico-chemical soil data were tested for deviation from normal distribution using a Kolmogorov-Smirnov test, and means for the three sampled sites were then compared employing a one-way ANOVA; significance of paired-comparisons was afterwards assessed using a Tukey post-hoc test. Univariate ANOVA was performed for testing the effects of multiple factors on the seed production and seed germination data. We used a three-factor ANOVA to test the effects of three factors on seed production: 1) Year (2011/2014); 2) pollen source (same species/different species); 3) mother species (*R. cyanocarpum*/*R. delavayi*). For seed germination data, we first analyzed the data from the germination in distilled water using a two-factor ANOVA to test effects of pollen source and mother species on seed germination; then a three-factor ANOVA was employed to examine the effects of three factors (soil type, pollen source and mother species) on seed germination. After the confirmation of no effect on seed germination by soil type, a combined analysis of water and soil treatments for seed germination was done using a three-factor ANOVA (medium, pollen source and mother species). All statistical analyses were performed using SPSS 15.0 for Windows (SPSS, Chicago, IL, USA).

## Additional Information

**How to cite this article**: Ma, Y.-P. *et al*. Strong reproductive isolation despite occasional hybridization between a widely distributed and a narrow endemic *Rhododendron* species. *Sci. Rep*. **6**, 19146; doi: 10.1038/srep19146 (2016).

## Supplementary Material

Supplementary Information

## Figures and Tables

**Table 1:** Equations used to quantify components of reproductive isolation.

Barrier	Equation for calculating RI
Prezygotic	
Geographic	1 – (no. of counties in which the species co-occur/no. of counties in which the species occurs)
Phenology	1 – (no. of days in which both species are in flower at the same time/no. total flowering days for the species)
Pollinator assemblage	1 – (flower visits by shared pollinators/total number of flower visits)
Pollinator behavior	1 – (no. of flower visits by pollinators coming from the respective other species/total no. of flower visits)
Postzygotic	
Pollen precedence	1 – (the ration of pollen tube length to the total length of the style from interspecific/the ration from intraspecific pollination)
Seed production	1 – (no. of seeds per interspecific/no. seeds per intraspecific pollination)
Seed germination	(1 – no. of germinated seeds resulting from interspecific pollination/no. germinated seeds resulting from intraspecific pollination)

Each RI value was generally varying from 0 to 1, with 0 representing no barrier to inter-specific gene flow and 1 representing a complete barrier.

**Table 2:** Volatile compounds extracted from flowers of *R. cyanocarpum* and *R. delavayi*.

Species	R. *delavayi*			R. *cyanocarpum*		
	Relative content (%)	Absolute production (ng/h/flower)	Absolute production (ng/h/inflorescence)	Relative content (%)	Absolute production (ng/h/flower)	Absolute production (ng/h/inflorescence)
Aliphatics	56.2	38.26	726.94	64.37	595.98	3575.88
Terpenoids	11.04	7.52	142.88	29.88	276.65	1659.9
Benzenoids	3.14	2.14	40.66	2.48	22.96	137.76
Total	70.38	47.91	910.29	96.71	895.41	5372.46

Shown are the relative contribution of compound groups to the total mass, and the absolute production per hour per flower and average production per hour per inflorescence.

**Table 3:** ANOVA results for the effect of the factors (year, pollen source, and mother species) on the number of seeds produced.

Factors	Sum of Squares	df	Mean Square	F	p
Year	425756.64	1	425756.64	5.03	0.03
Pollen source	1806197.96	1	1806198	21.33	<0.001
mother	2504887.08	1	2504887.1	29.58	<0.001
Year * Pollen source	86980.96	1	86980.96	1.03	0.31
Year * mother	107904.48	1	107904.48	1.27	0.26
Pollen source * mother	525313.38	1	525313.38	6.20	0.01
Year * Pollen source * mother	128169.48	1	128169.48	1.51	0.22

**Table 4:** Soil physicochemical properties of samples taken from three different localities (one *R. delavayi* dominated population, one *R. cyanocarpum* dominated population, and the hybrid zone).

Site	R. *delavayi dominated*	R. *cyanocarpum dominated*	Hybrid zone
Altitude (m)	2800 ~ 3000	3200 ~ 4000	3100
pH	4.97 ± 0.05^a^	4.02 ± 0.04^b^	4.48 ± 0.1^c^
Organic matter (g/kg)	210.42 ± 3.78^a^	257.35 ± 3.50^b^	234.56 ± 1.17^c^
Total nitrogen (g/kg)	7.73 ± 0.13^a^	8.97 ± 0.09^b^	9.92 ± 0.56^b^
Total phosphorus (g/kg)	1.16 ± 0.03^a^	1.24 ± 0.04^a^	1.42 ± 0.06^b^
Total potassium (g/kg)	2.92 ± 0.02^a^	1.85 ± 0.08^b^	3.64 ± 0.10^c^
Available phosphorus (g/kg)	18.14 ± 0.41^a^	33.88 ± 2.07^b^	34.86 ± 7.32^b^
Available potassium (g/kg)	276.18 ± 2.97^a^	303.65 ± 5.25^a^	243.10 ± 42.76^a^

Values with different letter superscripts are significantly different on the p < 0.05 level.

**Table 5:** ANOVA results testing for the effect of the factors medium (water vs soil), pollen source, and mother species on the seed germination rate of seed obtained from four different pollination treatments.

Source of variation	Sum of Squares	d.f.	F	*p*
Different soil treatments				
Soil type	0.014	2	2.311	0.115
Pollen source	0.258	1	83.814	0
Mother	0.652	1	211.702	0
Pollen source * Mother	0.121	1	39.376	0
Soil type * Pollen source	0.001	2	0.158	0.854
Soil type * Mother	0.003	2	0.409	0.667
Soil type * Pollen source * Mother	0.007	2	1.181	0.319
Distilled water treatment				
Pollen source	0.055	1	38.145	0
Mother	1.519	1	1060.300	0
Pollen source * Mother	0.002	1	1.308	0.286
Combined analysis of soil and water germination				
Medium	0.874	1	307.989	0
Pollen source	0.217	1	76.542	0
Mother	2.079	1	732.960	0
Medium * Pollen source	0.003	1	0.922	0.342
Medium * Mother	0.555	1	195.572	0
Pollen source * Mother	0.010	1	3.400	0.071
Medium * Pollen source * Mother	0.031	1	10.800	0.002

**Table 6:** Contribution of assessed barriers to RI between *R. cyanocarpum* and *R. delavayi*.

Isolating barrier	Components of RI		Absolute contributions to total RI		In sympatry (pre-pollination)	
	*R. cyanocarpum*	*R. delavayi*	*R. cyanocarpum*	*R. delavayi*	*R. cyanocarpum*	*R. delavayi*
Geographic	0	0.98	0	0.98	–	–
Phenology	0.043	0.141	0.043	0.0028	0.043	0.141
Pollinator assemblage^1^	0.016	0.365	0.015	0.006	0.015	0.316
Pollinator behavior^2^	1	1	0.942	0.0112	0.942	0.543
In total			1	1	1	1
					In sympatry (post-pollination)	
Pollen precedance^3^	0.281	0.557	0	0	0.281	0.557
Seed production^4^	0.775	0.15	0	0	0.557	0.066
Seed germination^5^	0.627	0.833	0	0	0.102	0.314
In total			0	0	0.94	0.937

^1^Average RI for data obtained in 2008 and in 2011.

^2^RI = 1 due to no observed transfer visits. As hybrids do occur in the wild this is obviously an overestimate, but might be correct for certain years.

^3^RI obtained by extrapolating data, likely to be a very rough estimate.

^4^Averaged from data obtained in 2011 and 2014.

^5^RI as calculated for seed germination in soil, which better reflects natural conditions.

## References

[b1] Jennings J. H., Snook R. R. & Hoikkala A. Reproductive isolation among allopatric *Drosophila* Montana populations. Evolution 68, 3095–3108 (2014). 25302639 10.1111/evo.12535

[b2] Schemske D. W. Adaptation and the origin of species. Am. Nat. 176(**S1**), S4–S25 (2010). 10.1086/657060 21043779

[b3] Nosil P. & Feder J. L. Genomic divergence during speciation: causes and consequences. Philos. Trans. R. Soc. B Biol. Sci. 367, 332–342 (2012). 10.1098/rstb.2011.0263 PMC3233720 22201163

[b4] Ramsey J., Bradshaw H. D. & Schemske D. W. Components of reproductive isolation between the monkey flowers *Mimulus lewisii* and *M. cardinalis* (Phrymaceae). Evolution 57, 1520–1534 (2003). 12940357 10.1111/j.0014-3820.2003.tb00360.x

[b5] Kay K. M. Reproductive isolation between two closely related hummingbird pollinated neotropical gingers. Evolution 60, 538–552 (2006). 16637499

[b6] Dell’Olivo A., Hoballah M. E., Gübitz T. & Kuhlemeier C. Isolation barriers between *Petunia axillaris* and *Petunia integrifolia* (Solanaceae). Evolution 65, 1979–1991 (2011). 21729053 10.1111/j.1558-5646.2011.01279.x

[b7] Baack E., Melo M. C., Rieseberg L. H. & Ortiz-Barrientos D. The origins of reproductive isolation in plants. New Phytol. 207, 968–984 (2015). 25944305 10.1111/nph.13424

[b8] Chamberlain D. F. A revision of *Rhododendron* II. Subgenus *Hymenanthes*. Not. R. Bot. Garden Edinburgh 39, 209–486 (1982).

[b9] Fang R. Z. & Min T. L. The floristic study on the genus Rhododendron. Acta Botanica Yunnanica 17, 359–379 (1995).

[b10] Zhang J. L., Zhang C. Q., Gao L. M., Yang J. B. & Li H. T. Natural hybridization origin of *Rhododendron agastum* (Ericaceae) in Yunnan, China: inferred from morphological and molecular evidence. J. Plant Res. 120, 457–463 (2007). 17393071 10.1007/s10265-007-0076-1

[b11] Ma Y. P., Milne R. I., Zhang C. Q. & Yang J. B. Unusual patterns of hybridization involving a narrow endemic *Rhododendron* species in Yunnan (Ericaceae), China. Am. J. Bot. 97, 1749–1757 (2010a). 21616807 10.3732/ajb.1000018

[b12] Ma Y. P., Zhang C. Q., Zhang J. L. & Yang J. B. Natural hybridization between *Rhododendron delavay*i and *R. cyanocarpum* (Ericaceae), from morphological, molecular and reproductive evidence. J. Integr. Plant Biol. 52, 844–851 (2010b). 20738728 10.1111/j.1744-7909.2010.00970.x

[b13] Zha H. G., Milne R. I. & Sun H. Asymmetric hybridization in *Rhododendron agastum*: a hybrid taxon comprising mainly F_1_s in Yunnan, China. Ann. Bot. 105, 89–100 (2010). 19887474 10.1093/aob/mcp267 PMC2794068

[b14] Marczewski T., Chamberlain D. F. & Milne R. I. Hybridization in closely related *Rhododendron* species: half of all species-differentiating markers experience serious transmission ratio distortion. Ecol. Evol. doi: 10.1002/ece3.1570 (2015). PMC4559045 26357534

[b15] Milne R. I., Davies C., Prickett R., Inns L. H. & Chamberlain D. F. Phylogeny of *Rhododendron* subgenus *Hymenanthes* based on chloroplast DNA markers: Between lineage hybridisation during adaptive radiation? Plant Syst. Evol. 285, 233–244 (2010).

[b16] Milne R. I., Terzioglu S. & Abbott R. J. A hybrid zone dominated by fertile F_1_s: maintenance of species barriers in *Rhododendron*. Mol. Ecol. 12, 2719–2729 (2003). 12969475 10.1046/j.1365-294x.2003.01942.x

[b17] Johnson M. A., Price D. K. & Stacy E. A. Postzygotic barriers isolate sympatric species of *Cyrtandra* (Gesneriaceae) in Hawaiian montane forest understories. Am. J. Bot. 102, 1870–1882 (2015). 26542848 10.3732/ajb.1500288

[b18] Fang Q., Chen Y. Z. & Huang S. Q. Generalist passerine pollination of a winter-flowering fruit tree in central China. Ann. Bot. 109, 379–384 (2012). 22112440 10.1093/aob/mcr293 PMC3268538

[b19] Briscoe A. D. & Chittka L. The evolution of color vision in insects. Annu. Rev. Entomol. 46, 471–510 (2001). 11112177 10.1146/annurev.ento.46.1.471

[b20] Knudsen J. T., Eriksson R., Gershenzon J. & Stahl B. Diversity and distribution of floral scent. Bot. Rev. 72, 1–120 (2006).

[b21] Ma Y. P., Wu Z. K., Dong K., Zhang C. Q., Sun W. B. & Marczewski T. Pollination biology of *Rhododendron cyanocarpum* (Ericaceae), an alpine species endemic to NW Yunnan, China. J. Sys. Evo. 53, 63–71 (2015).

[b22] Wesselingh R. A. & Arnold M. L. Pollinator behavior and the evolution of Louisiana iris hybrid zones. J. Evo. Biol. 13, 171–180 (2000).

[b23] Lowry D. B., Modliszewski J. L., Wright K. M., Wu C. A. & Willis J. H. The strength and genetic basis of reproductive isolating barriers in flowering plants. Philos. Trans. R. Soc. B Biol. Sci. 363, 3009–3021 (2008). 10.1098/rstb.2008.0064 PMC2607309 18579478

[b24] Gibbs D., Chamberlain D. F. & Argent G. The Red List of Rhododendrons. Botanic Gardens Conservation International, Richmond, p 128 (2011).

[b25] Fang Q. & Huang S. Q. Relative stability of core groups in pollination networks in a biodiversity hotspot over four years. Plos one 7, e32663 (2012). 22412902 10.1371/journal.pone.0032663 PMC3297609

[b26] Liu C. Q. & Huang S. Q. Floral divergence, pollinator partitioning and the spatiotemporal pattern of plant–pollinator interactions in three sympatric *Adenophora* species. Oecologia 173, 1411–1423 (2013). 23824141 10.1007/s00442-013-2723-7

[b27] Hou X., Duan C., Tang C. Q. & Fu D. Nutrient relocation, hydrological functions, and soil chemistry in plantations as compared to natural forests in central Yunnan, China. Eco. Res. 25, 139–148 (2010).

