# Supplementary material for: Strong reproductive isolation despite occasional hybridization between a widely distributed and a narrow endemic Rhododendron species
Source: Sci Rep. 2016 Jan 11;6:19146. doi: 10.1038/srep19146 (PMC4707479; doi:10.1038/srep19146)
Supplement: Supplementary Information [file srep19146-s1.pdf]

Strong reproductive isolation despite occasional hybridization between a widely distributed and a narrow endemic *Rhododendron* species

Yong-Peng Ma, Wei-Jia Xie, Wei-Bang Sun & Tobias Marczewski

Table S1 Volatile compounds detected in extracts from flowers of *Rhododendron cyanocarpum* and *R. delavayi*

						R. cyanocarpum		*R. delavayi*	
Compound	CAS	tn	tn+1	RT	RI	Relative content (%)	Absolute production	Relative content (%)	Absolute production
Propyl acetate	109-60-4	1.92	2.38	3.69	884.57	1.19	10.97		
3-hexanone	589-38-8	2.38	3.42	5.10	861.44	0.81	7.47	1.80	1.22
2-hexanone	591-78-6	2.38	3.42	5.21	872.21	0.95	8.76	1.83	1.25
3-hexanol	17015-11-1	2.38	3.42	5.37	887.12	0.98	9.10	1.75	1.19
n-hexaldehyde	66-25-1	2.38	3.42	5.46	896.35	2.26	20.93	3.69	2.51
Diacetone alcohol	123-42-2	2.38	3.42	6.69	1014.04	15.39	142.51	14.80	10.08
α -Pinene	80-56-8	12.97	17.2	10.01	929.62	4.08	37.82	2.78	1.89
Sabinene	3387-41-5	12.97	17.2	11.72	970.19	4.12	38.14		
methyl heptenone	110-93-0	5.62	8.91	12.38	1005.50	6.27	58.03	5.95	4.05
octanal	124-13-0	5.62	8.91	13.09	1027.05	2.25	20.83	1.54	1.05
Eucalyptol	470-82-6	12.97	17.2	14.27	1031.00	6.08	56.32	1.71	1.16

Ocimene	13877-91-3	12.97	17.2	15.08	1050.24	2.91	26.92		
Linalool	78-70-6	12.97	17.2	17.18	1100.31	10.00	92.62	2.04	1.39
Nonanal	124-19-6	8.91	13	17.31	1106.95	10.85	100.45	8.14	5.54
Tridecanal	10486-19-8	23.76	26.6	19.66	1153.05	2.53	23.38	1.50	1.02
Bihexyl	112-40-3	20.69	23.8	20.64	1198.37	3.80	35.15	1.98	1.35
Decanal	112-31-2	12.97	17.2	20.86	1187.86	12.77	118.21	11.15	7.59
n-tetradecane	629-59-4	26.55	29.1	26.51	1398.44	1.41	13.06	2.07	1.41
β -selinene	18423-23-9	29.12	31.5	28.94	1492.53	4.63	42.84	1.92	1.30
α -selinene	473-13-2	29.12	31.5	29.16	1501.45	0.97	8.96	2.60	1.77
2,6-Di-tert-butyl-4-methylphenol	128-37-0	29.12	31.5	29.52	1516.60	2.48	22.98	3.06	2.08
Aliphatics						64.37	595.98	56.20	38.26
Terpenoids						29.88	276.65	11.04	7.52
Benzenoids						2.48	22.96	3.14	2.14
Unknowns						3.29	30.46	29.62	20.16
Total						100	925.87	100	68.07

Kovats index (KI) was calculated according to the formula of Van Den Dool & Kratz (1963) as given in Chen et al. (2012): KI=100 n + 100(t_x -t_n)/(t_{n+1}-t_n), where n is the number of carbon atoms in the *n*-alkane eluting immediately before the compound of interest. CAS, Chemical Abstracts Service registry number; t_n, retention times of the *n*-alkanes eluting immediately before; t_{n+1}, retention times of the n-alkanes eluting immediately after; *RT*, retention time of the compound.
